# Scientific Items

**Published:** 1885-11-20

**Authors:** 


### SCIENTIFIC ITEMS.

**Another New Metal?**—One comes to be distrustful of "alleged" new metals, so often do they fail to secure a permanent place in the list of so-called "elements;" but we feel bound to chronicle the announcement of each fresh claimant for the honor. Norwegium is the name given to one which Dr. T. Dahll believes he has discovered in examining a specimen of nickel ore from Krageroe, in Norway. It is a malleable metal, of white color with a tinge of brown. It presents, when pure, a metallic lustre, but on exposure to the atmosphere becomes coated with a thin film of oxide. Its hardness is about that of copper, and its specific gravity is 9.4441. At 350°C. it melts. From its physical properties and chemical reaction, it appears to differ from every other known metal; and Dr. Dhall claims for it a distinct individuality.—*Popular Science News.*

**Our Country** is setting a good example to Europe in this recognition of the fact that the grand and beautiful works of nature should be made national property. The preservation of the Yosemite Valley and of the Yellowstone district will redound to the everlasting honor of a people that has been suspected of overweening devotion to the "almighty dollar." They show that the Yankee race is not altogether selfish, and that, in its pursuit of the real and the practical, it has not become entirely insensible to the claims of the ideal and the poetical. While it sets the falling streams to work, in turning the wheels of industry, it can rescue the noblest of its cataracts from that degradation, in order that it may remain "a thing of beauty," and thus be "a joy forever" to the lovers of nature. Let us hope that our friends on the other side of the Niagara River may supplement the good work by a similar "reservation" of the Canadian shores near the falls. It is said that there is a lack of zeal in behalf of the movement inaugurated with this view, but we are unwilling to believe that the apathy is other than transient. Canada cannot afford to let New York outdo her in this honorable enterprise.—*Ib.*

**Experiments on the Decapitated.**—The question has again been mooted in French scientific circles, whether those who have been decapitated suffer: and after additional experiments on the head of Gamahut, who was lately guillotined on the 24th of Aug., 1885, for the murder of a widowed lady in Paris, coupled with those related by Claude Bernard, the presumption is that they do; for, under the influence of transfused blood, the blanched features recovered almost their normal expression, the eyelids were slightly open, and the lips quivered for a few seconds, as if to express some perception communicated from the brain. The conclusion, then, is, that, so long as the brain contains any blood, the head of the decapitated person, which falls into a receptacle prepared for the purpose, is capable of seeing, of hearing, and of knowing that it is separated from the body. This view, however, was repudiated by Professor Vulpian at the last meeting of the Academy of Medicine, on the following grounds: a strong blow applied to the head, or a less severe one to the stomach, would cause instantaneous syncope; that is to say, consciousness would for the moment be entirely abolished. So it would be in the case of the severed head, but of course without any possibility of recovery: the weight of the guillotine falling on the neck would produce the same result. Moreover, syncope would be occasioned by the sudden division of the carotid arteries, and the consequent emptying of the arterial system of the brain. Therefore, at the very moment that the brain ceases to receive the vivifying fluid from the heart, it loses all power of excito-motility, as well as all power of sensation: all this transpires as quickly as thought, and its duration may be compared to a flash of lightning.—*Ib.*

**A Paper Chimney.**—A manufacturer of Breslau is stated to have built a chimney, over 50 feet in height, entirely of paper. The blocks used in its construction, instead of being of brick or stone, were made of compressed paper, jointed with silicious cement. The chimney is said to be very elastic, and also fireproof. We may add that picture frames are now made of paper on the Continent. Paper pulp, glue, linseed oil, and carbonate of lime or whiting are mixed together and heated into a thick cream, which, on being allowed to cool, is run into moulds and hardened. The frames are then gilt or bronzed in the usual way.—*Scientific American.*

**Electricity and Dust.**—To say that new applications of electricity are being found almost daily, is mere common place; and yet we cannot help being surprised at some of the novel uses to which this marvelous agent is coming to be put. We have only begun to learn its powers and capabilities. If we could look a hundred years into the future, no doubt we should be as much amazed at the part that electricity will then be playing in the economy of every-day life, as the man of a century ago would be at the application of steam nowadays to manufacturing purposes, and to locomotion on land and water.

Electricity may yet be of important service as a sanitary agent. It is only within a short time that the discovery has been made that a discharge of electric sparks through a dusty atmosphere will cause the dust to settle, and already the fact is beginning to yield results of practical value.

In the experiments to which we have referred, a continuous series of sparks were sent through a bell-jar filled with magnesium smoke; and in a very short time the air in the jar was cleared: similarly, when the jar was filled with steam, electrification caused the globules to aggregate into mist and fine rain, which rapidly fell.—*Popular Science News.*

**Dyed** tomatoes have been offered for sale in Paris. The fruit not having acquired its usual deep hue, the producers covered it with red paint, for which, on discovery, they were fined a hundred francs.

